# A Brief Report of an Epidemic Erysipelas, Which Occurred in South Otselic and Vicinity, Chenango Co., N. Y.

**Published:** 1854-11

**Authors:** 


ART. III.—*A Brief Report of an Epidemic Erysipelas, which occurred in South Otselic and vicinity, Chenango Co., N. Y., during the winter and spring of* 1854. By Drs. AVERY & JAMESON.

During the latter part of the winter and spring of 1854, South Otselic and neighborhood experienced a severe visitation of epidemic erysipelas. Prior to this time, this district had been remarkable for its general salubrity, and, up to the period of the epidemic under consideration, erysipelas was a disease but little known, only a very few sporadic cases having been met with. The winter was peculiar on account of the comparatively little snow which fell, and the great, sudden, and frequent changes in the weather—heavy rains being quickly followed by hard frost and severe cold, which, however, was of brief duration; so that, on the whole, the season was decidedly milder than usual. As early as Nov. 29, 1853, we were requested to visit a patient who, after exposure to the cold night air whilst traveling, was seized with the usual symptoms of a violent cold, for which he took a sweat with advantage, yet did not feel really well; and on going out, took additional cold, for which sweating was again resorted to with benefit; some fever, however, still continuing, was the reason of our being called in. It is only necessary to state, that the patient passed through a mild course of fever which appeared to terminate by a copious diaphoresis—that he did not make any complaint of his throat, further than that it felt dry; and when examined, the mucous membrane of the fauces looked considerably red, but there was no appearance of effused lymph, no ulceration, tonsils but little tumefied, and no swelling externally.

When this patient began to be convalescent, a young lady residing in the same house, was attacked with sore throat of a severe character, attended with some swelling and tenderness externally; slight enlargement of the tonsils; ulceration of the mucous membrane; and, probably, the formation of one or more abscesses in the neighborhood of the tonsils, and which broke internally. The nitrate of silver was freely applied, three or four times, to the fauces; and, together with laxatives and nutritious diet, sufficed for the recovery. We did not, at the time, have any suspicion of there being anything erysipelatous in either of the above cases; neither do we now positively affirm such to be the case; and they are introduced here chiefly on account of their connection with the next case, concerning the erysipelatous character of which, we consider, there can be no doubt. The patient, first mentioned, was visited, during his sickness, by his relative, Mrs. P., and early on the 4th of January, 1854, being a short time after her visit to her brother, we were desired to see her. The patient informed us, that the menses had been flowing for the past week, and that, during this period, her throat had been very painful, accompanied with frequent cold chills, for which she had taken a sweat, but had not confined herself to the house or bed; that her throat was very sore on going to bed last night, so that she could only swallow with difficulty and pain; and the bowels being rather constipated, she deemed it best to take a dose of salts, which operated violently in the course of the night, the throat feeling very much better, but a copious hæmorrhage taking the place of the ordinary menstrual flow. The following is a brief account of the symptoms on this morning, as extracted from notes of the case: "Copious discharge of blood per vaginam, causing some faintness; skin a little warmer than natural; pulse 100, and rather small; tongue clean, dry, and glazed; says her tongue has felt sore, as if raw, for several days; fauces look considerably red, but no ulceration, or particular tumefaction of the tonsils, and no swelling externally." She did not complain of any pain in the abdomen, and our attention was not directed to it, being absorbed with a consideration of the hæmorrhage, for which the superacetate of lead and opium was ordered. Without entering into the minutiæ of the case, it may suffice to state, that on visiting the patient again in the evening, we found her better, so far as the hæmorrhage was concerned, but complaining of pain in the abdomen, which was considerably tender on pressure in the hypogastric region; and that intense and universal peritonitis was quickly established, ending fatally forty-eight hours from the time of our first visit.

On inspection of the body, we found the peritoneum universally inflamed, the small intestines matted together; and in the peritoneal sac, a copious effusion of sero-flocculent fluid combined with pure pus, the latter being especially abundant in the pelvic cavity. This, we believe, was the first decided case of erysipelatous fever and inflammation that appeared in our neighborhood; and the next case in succession occurred in a relative of Mrs. P's, who arrived at her house, from a distance of twenty miles, either the day of her death, or the day after, and began to be unwell a few days subsequently, but did not confine herself to bed until about a fortnight afterward, when our attendance was requested. It is only necessary to remark here, that the patient passed through a regular attack of erysipelas of the head and face, terminating favorably. We may state that this and the former case were both seen in consultation, by Dr. Bowen, of Pitcher. From this time, the erysipelas became general, the patients being distant from each other, and having had no communication with any suffering from the disease, rendering it manifest to us, that the peculiar and unknown condition of the atmosphere, designated in our ignorance as erysipelatous, prevailed; and that we had to grapple with a more than ordinary disease.

We have been thus minute, perhaps unnecessarily so, in relation to the rise of the epidemic, both in order that our readers may form some conception as to the manner of its commencement, and as affording an interesting illustration of the insidious mode in which such epidemics may sometimes be developed. In proceeding to give a particular description of the disease, as it occurred to our observation, the different cases seem naturally to arrange themselves under the five following heads or divisions:

1. Those characterized by sore throat and febrile symptoms, but in which there was no external manifestation of erysipelas, nor any internal inflammation.

2. Those in which erysipelas appeared externally, and most commonly on the face and head, being generally preceded by fever and sore throat.

3. Those marked by the occurrence of some internal inflammation, and generally preceded, for a longer or shorter period, by sore throat and fever, but rarely by erysipelas externally.

4. Those distinguished by the enlargement of one or more glands in the region of the axilla, preceded by general malaise, and more or less fever; and, after a variable period, ending in suppuration.

5. The ordinary phlegmonous erysipelas, commencing in the hand or foot, and involving, not unfrequently, to an alarming, if not fatal extent, other and remote parts of the system.

It is scarcely necessary to observe, that we do not regard the above arrangement as indicative of so many distinct diseases, but as merely varieties of one and the same disease, recognizing the same remote and exciting causes, demanding the same principle of treatment, and differing only, in consequence of occult constitutional dispositions, or other unknown causes, in the mode of manifestation.

The invasion of the disease, in some of our patients, was characterized by slight soreness of the throat, stiffness and pain of the neck, more or less general malaise, and, in the course of a few hours, the supervention of cold chills; in others, and more especially, (as was not unfrequently the case,) where the patients had been exposed to cold, the chills were nearly simultaneous with the occurrence of sore throat, pain of the head, &c. In whichever of these modes the disease commenced, the rigors were sometimes remarkably violent, and, when so, generally a premonition of the subsequent severity of the case. The cold chills or rigors were accompanied by pain in the back, head, and limbs, the two former being sometimes particularly distressing, and by a more or less coated state of the tongue. These symptoms were followed, sooner or later, by fever more or less violent; increasingly painful condition of the throat; swelling and tenderness in the region of the tonsils, or parotid and submaxillary glands; pains, apparently muscular, at the back and sides of the neck; great pain and difficulty in deglutition; and, in some cases, an almost total inability to open the mouth. On examination of the fauces, where it could be done, there was found to be more or less redness of the mucous membrane and tumefaction of the tonsils; but, it is worthy of remark, that the pain and difficulty of deglutition were frequently greater than the condition of the tonsils, or inflammation of the mucous membrane, would seem to account for, and appeared best explained by the inflammation and swelling of the parotid and submaxillary glands, conjoined, perhaps, with an irritable and spasmodic state of the adjacent muscles. The swelling, at the root of the ear, was undoubtedly dependent, in many of the cases, upon inflamed and enlarged tonsils; but, in others, the tumefaction was too great and diffused to be ascribed to this cause, and was evidently connected with a swollen and inflamed condition of the parotid and submaxillary glands. The above symptoms varied greatly in severity, and the subsequent progress of the cases was equally variable. In some, the patients were speedily relieved by passing through a copious diaphoresis, sinapisms to the throat, laxatives, and attention to diet, being convalescent in a few days; whilst, in others, these means afforded only partial relief, erysipelas manifesting itself on the face or elsewhere, some internal inflammation taking place, or the fever was protracted for two or three weeks without any apparent internal complication.

In none of the cases observed by us did the external swelling of the throat proceed to suppuration, although, in one severe case, there appeared to be eminent danger of it; and about five weeks elapsed before the tumefaction entirely disappeared. There are two circumstances here, appearing to us worthy of a moment's consideration:

1. The manner in which the erysipelas first made its appearance on the surface.

2. At what period from the first manifestation of febrile symptoms.

In relation to the first, namely, the mode of development of the erysipelas, there did not appear to be any uniformity. In two cases, the cutaneous inflammation had its starting point in the swelling of the parotid gland, spreading from thence, in one of them, over the posterior part of the head to the forehead and face; and, in the other, to the face and forehead in the first instance. In a third, the morbid action seemed to be distinctly concentrated on one of the axillary glands, which was enlarged and exquisitely painful, but did not suppurate; from this gland the erysipelas spread down the whole of the same side until it arrived at the lumbar region, where it terminated in diffused suppuration, followed by death of the patient. Lastly, and most commonly, it manifested itself on the face by a redness across the bridge of the nose, or by redness and tumefaction of the end of the nose and upper lip, having spread from the mucous membrane of the fauces to the Schneiderian membrane, and so creeping along to the face.

In regard to the second consideration, namely, the period elapsing from the first manifestation of fever to the external development of erysipelas, there did not seem to be anything definite. In one case, that of Mrs. C., to which we were called on the 24th of Feb., the day of attack, which was very severe, her throat, especially on the right side, being then so swelled externally that the teeth could only be separated very slightly, it was not until the 6th of March, ten days afterward, that erysipelas appeared on the swelling of the right side, and diffused itself from thence over the face. This was the longest interval occurring to our observation, the shortest being about three days.

We pass now to the consideration of one of the most interesting and important features of the disease, namely, the internal inflammations with which it was complicated. One patient had laryngitis, of which he died. Another bronchitis, also fatal. Three pleuro-pneumonia, and recovered. Two died apparently from cerebral inflammation. Seven were attacked with peritonitis, all of whom died, except one. Besides these seven cases which fell under our own observation, we heard of three or four other fatal cases occurring in the practice of neighboring physicians, from which it is evident, that by far the most numerous and fatal of the complications, in this epidemic, was peritonitis. One of the above cases of peritonitis occurred in a woman who had only been delivered a few days, and exhibited all the characteristics of puerperal peritonitis, ending fatally in forty-eight hours. We attended the patient in her labor, and the case may, perhaps, be adduced as additional confirmation of the supposed identity, of one form at least, of puerperal peritonitis with erysipelas. It may be proper to state, however, that there were other cases of parturition in the neighborhood during the prevalence of the epidemic, but all the women, we believe, passed favorably through the period of their accouchments.

So far as our very limited reading extends, but little has, we believe, been written on the subject of Erysipelatous Peritonitis, a brief chapter in the work of Abercrombie, on the Stomach and Bowels, being the most extended notice of the disease that we have met with. Supposing some of our readers to be as ignorant as ourselves, prior to the epidemic under consideration, in reference to one of the most iusidious and fatal diseases which the physician has to contend with, we shall gladly communicate the fruit of our experience, in reference to one or two points not sufficiently elucidated by any writer with whose productions we are familiar. And, 1st. The frequent commencement of the inflammation subsequent to the administration of a purgative. In one of the cases related by Abercrombie, abnormal pain supervened almost immediately upon the operation of laxative medicine, the patient dying in forty-eight hours from the commencement of the attack. The case of Mrs. P., already alluded to in the beginning of our report, appears to us very interesting in relation to this point, being like that of Dr. Abercrombie's, confirmed by a *post-mortem* examination. It will be remembered that, after taking a dose of salts, Mrs. P. retired to bed on the Tuesday night, with the throat very sore and the menses flowing; and that in the course of the night the medicine operated severely, the throat being much relieved, but a copious hæmorrhage taking place per vaginam. Her condition, when seen by us about six o'clock on Wednesday morning, has already been presented; and, to our own minds, there is no doubt as to the peritonitis having then commenced, and that both it and the hæmorrhage may reasonably be ascribed to the irritation of the salts.

In the second case to which we were called, the patient being a little boy about five years of age, the mother of the child informed us, that on the Saturday his throat was very sore and swelled externally, for which she used simple remedies with advantage. On the following day she gave him a dose of salts, which acted freely on the bowels, their operation being speedily followed by pain in the abdomen, and vomiting, the matter ejected from the stomach being of a *green* color. Without entering into the details of the case, it will be sufficient to state that the usual symptoms of peritonitis were present, and terminated fatally about fifty-six hours from the time of taking the salts.

In a third case, which we saw in consultation, calomel and blue pill had been given as a purgative, followed by pain in the abdomen of a griping character, for which morphine was ordered. When seen by us on the following morning, the patienr exhibited all the symptoms of intense and universal peritonitis, and died in thirty hours.

In a fourth case, the symptoms followed the administration of a full dose of ol. ricini; and, in this instance, something like metastasis to the brain occurred, the patient being violently delirious during the last twelve hours of her life.

We have here, then, five cases of erysipelatous fever and sore throat, in which the operation of a purgative was speedily followed by intense and fatal peritonitis; and, although they may not be sufficiently numerous to justify the deduction of a general principle, they are, nevertheless, sufficient, we should suppose, to convince us of the propriety of exercising much caution in relation to the use of purgatives in erysipelatous fever. We do not undertake to demonstrate how a purgative operates to light up abdominal inflammation in a patient laboring under this disease, whether by that mysterious phenomenon, metastasis, or in some other way; but the above-cited facts seem to justify us in believing, that a patient who is the subject of this fever will, if he takes a cathartic, be in greater danger of being attacked with peritonitis, than if the bowels were let alone, or only a very gentle laxative administered. It would seem, however, as though during the existence of this fever, any local irritation, or increased flow of blood to any part or organ, incurred eminent hazard of establishing inflammation in such part or organ.

2. The frequently insidious origin, and sometimes, progress of the inflammation. So much is this the case, and the time so brief in which treatment can be of any avail, that, from the limited observation we have had, we should be disposed to consider that there are but few physicians who would not, unless particularly well informed as to the nature of the disease, and the mind fully awake to its existence, be very likely to fail in the diagnosis of the first case they might encounter, and thereby lose the patient. The pain may readily be confounded with a griping occasioned by a previous dose of physic; and morphine or opium in some form administered, by which the patient may, perhaps, be lulled to sleep, but it only leads too surely to the sleep of death. There is nothing of which we feel more the importance, than of a prompt and careful manipulation of the abdomen, on the occurrence of the slightest pain, or any other symptom of a suspicious character. 3. The rapidly fatal character of the inflammation. This is a marked feature of the disease, and in which it bears much resemblance to puerperal peritonitis. In the course of a few hours the whole of the peritoneum will be involved in the inflammation, and the fate of the patient almost inevitably sealed. In view of this frightful rapidity, how important that an early and correct diagnosis should be instituted, and a bold and efficient plan of treatment speedily adopted.

Before closing our remarks upon this part of the subject, we desire to add a few words in relation to two symptoms, namely, vomiting and dyspnœa, which are very general attendants on acute peritonitis, however induced. With regard to the first mentioned symptom we believe it to be very commonly present; and, further, that the matter ejected from the stomach is, almost always, of a *green* color, being, what Andral designates, green bile. Of fifteen cases of acute peritonitis, from different causes, related by this accurate and philosophic observer, in his Clinique Medicalé, vomiting is stated as being present in ten of them; and of the remaining five, nausea was present in two. In all of these ten cases, except one in which the character of the vomited matter is not described, the fluid ejected from the stomach is represented as "a green bile," or "a porraceous bile." In his first observation we read: "De la bile, en petite quantité à la fois, élait à chaque instant vomie." This description coincides precisely with what we observed in a case to which we were called in consultation, by our friend Dr. Halbert, of Pitcher. The patient had a vessel constantly by her, and at short intervals ejected from the stomach, as if by regurgitation rather than vomiting, a mouthful of green bile. She did this twice while we were manipulating the abdomen.

Dyspnœa, in the form of a short and hurried respiration, is generally present in a greater or less degree, and becomes more and more marked as the disease progresses; being, not unfrequently, heaving or laboring, toward the end. It was especially remarkable in the case of Mrs. P., and might readily have led to the supposition of thoracic inflammation. We remember, when a student, attending a case of puerperal peritonitis, of which a brief memorandum is possessed, in which the dyspnœa was so urgent that we concluded inflammation of the lungs must certainly exist; but, on inspection of the body after death, they were found, as in Mrs. P.'s case, perfectly healthy, and the peritoneum universally inflamed with copious sero-flocculent effusion in its cavity. It is scarcely necessary to observe, that in the dyspnœa of peritonitis the respiration will be found chiefly thoracic, a careful look at the thorax and gentle touch of the abdomen being, therefore, all that are required to dissipate any doubts and establish a correct diagnosis. In view of the great fatality of our cases of peritonitis, it may be only justice to ourselves to state, that, in three of them we did not see the patients until the symptoms were so far advanced as to render their condition, in a great degree, helpless.

*Contagiousness of the Disease.*—We consider the evidence strongly corroborative of the affirmative; and the community imbibed the doctrine so firmly, that it became difficult, in some cases, to obtain adequate nursing and watching.

*Type of the Epidemic.*—We have no hesitation in representing it as decidedly phlogistic. The pulse was generally full, and, in many cases, strong. We abstracted blood from the arm in ten cases, the blood being buffed and cupped in all of them, and in some remarkably so. In four of these cases there was no evidence of any internal inflammation, yet the blood exhibited the same buffy coat; a satisfactory evidence, we consider, of the inflammatory nature of the disease. Bleeding was well borne, and generally attended with much relief to the symptoms.

*Treatment.*—Having expressed so decided an opinion in reference to the inflammatory type of the disease, our plan of treatment will readily be conjectured, and a lengthy description altogether unnecessary. We are frank to acknowledge that, in the commencement of the epidemic and before we had become properly awake to the phlogistic character of the disease we had to contend with, we erred in not adopting a sufficiently energetic and antiphlogistic mode of treatment.

1. *General Treatment.*—Sweating was generally resorted to in the first instance, the cold chills and rigors seeming naturally to demand such a proceeding; and, so far as our experience in concerned, the practice was attended with decided benefit, and not unfrequently complete relief, where it was done effectually and proper care observed for a few days afterward. If the disease was not removed, the distressing chills were dissipated and the patient rendered more comfortable. The perspiration exhaled a peculiarly disagreeable fœtor. Calomel, pil. hydr., or hydr. c. cretâ with ipecac. and morphine, or Dover's powder and ol. ricini, in mild doses as a laxative, were the medicines commonly prescribed; and, at a later period of the epidemic, if the symptoms did not speedily yield to these measures, and the case was sufficiently urgent to demand it, blood was abstracted from the arm to the extent the patient would bear. The loss of blood, in the few cases coming under our consideration, was attended with much relief, and if the fever was not extinguished, it was so quelled as to yield readily to a continuance of the mercurial until the specific effect of the medicine was obtained. In most of the cases the symptoms yielded to the use of mercury alone; but, even in those of a mild character, we found it not unfrequently necessary to continue the mercurial until the gums became affected, ere the disease would be wholly subdued. Alkalies constituted a very useful adjunct. Such was the system of constitutional treatment adopted by us; and, in which, on the occurrence of any similar visitation—similar, we mean, in relation to the type of the disease, we should place the utmost confidence; the only difference we should make being a more frequent abstraction of blood even in cases that might do well without it, but in which, we now believe, the cure would be expedited by depletion. In the cases complicated with internal inflammation, the same plan of treatment required to be energetically carried out. In the only case of peritonitis which we succeeded in saving, the patient was seen in three hours (a very important item in these cases, as regards the question of life or death) from the accession of abdominal pain, which was of a stinging, cutting character. She was immediately bled to faintness, the blood being buffed and cupped, and a dose of calomel and morphine administered. The patient experienced much relief; but in two hours, there being still considerable tenderness on pressure, and the pulse pretty full, blood, to the amount of about ten ounces, was further removed, there being no buff upon it on this occasion. The calomel and morphine was steadily continued until the mouth became sore, and blisters liberally applied on the abdomen. Her recovery was uninterrupted.

Early in the epidemic we were induced to make use of the newly proposed remedy, tinct. ferri muriatis, and the following is the result of our experience in four of the milder cases of the disease: One became delirious under its use; a second comatose; the third died comatose; and in the fourth, the fever was much aggravated, making a severe case out of a mild one. In each of these cases the iron was continued for two or three days, and then abandoned for the antiphlogistic treatment. However successful the medicine might have been in other epidemics, or in some sporadic cases, we were soon satisfied that it would not answer our purpose. How could it, indeed, with such an excess of fibrin as we have shown to have existed in the blood; a morbid condition of the vital fluid for the removal of which, so far as our limited knowledge extends, no treatment has been found more successful than the antiquated one of our forefathers—comprised in the practice of what they termed phlebotomy, and the use of that venerable article of the pharmacopeia—mercury, notwithstanding all the maledictions which their sapient descendants, in this enlightened day and generation, have seen good to heap upon it.

2. *Local Treatment, or that of the Throat.*—We frequently experience more trouble and less satisfaction in this part of the treatment, than in any other; and still remain somewhat undecided as to the most speedy and successful mode of management. Notwithstanding all the means resorted to, the throat, in some cases, would continue inflamed, painful, and swollen, day after day, erysipelas finally appearing on the face, or the throat gradually amending, and the patient becoming slowly convalescent. Mustard poultices were very generally applied externally, keeping up thereby as much counter-irritation as the patient could bear; and, whilst in some cases their use appeared to be beneficial, in others we were disposed to regard it as injurious. In the case of Mrs. C., already alluded to, and where the right side of the neck was much swollen, we thought them decidedly hurtful, and abandoned their use for the application of a cloth wet in warm spirit and water, and covered with oiled silk: a mode of dressing attended with the best effects in her case. And, on the occurrence of another such epidemic, we should be disposed to omit, in a great measure, the use of sinapisms, substituting for them either warm fomentations, or the dressing with oiled silk as above described. We heard of the common domestic remedy, lobelia and whiskey, being beneficially applied in some cases. The throat, in a large proportion of the cases, was swabbed internally with a strong solution of nitrate of silver; and, in how many of these the application had the effect of arresting the inflammation of the mucous membrane, and preventing its development on the face and head, we are unable to say; but that its use was beneficial in several of the cases, and did truly contribute to arrest the morbid action going on in the mucous membrane, we are fully satisfied. In a few cases, however, the erysipelas made its appearance on the face, notwithstanding the application of the nitrate, a circumstance by no means condemnatory, in our judgment, of the appropriateness of the remedy in all cases, and its real value in a large number of them. An individual may die of inflammation where bleeding has been practiced, but it would be unphilosophical to deny, on that account, the utility of bleeding as a remedy for inflammation, inasmuch as reason and ample experience both combine to prove the reverse. So the nitrate of silver may be the appropriate local remedy for erysipelatous inflammation of the fauces, and fail only in consequence of the incompleteness of its application, or some other cause of which we are ignorant. We should, therefore, deem it proper to resort to its use on any future occasion. In employing the remedy, it is of importance to cleanse the fauces of the slimy mucus which not unfrequently adheres to the mucous membrane in considerable quantity, and, if not removed, would prevent the solution from touching the inflamed surface.

In regard to local applications to the erysipelas of the face and head, the nitrate of silver failed to arrest the progress of the inflammation in the three or four cases in which it was applied, a result not exactly in accordance with the experience of Mr. Higginbottom, (see Braithwaite, part 28, p. 234,) and which may have arisen from its inefficient application. We think favorably of this theory, and should be disposed to give the practice a more thorough trial. If the erysipelas manifested a disposition to spread down the neck, we should certainly apply it, and with considerable hope of being successful in arresting its course, from the fact of having seen it completely succeed in doing so in the lower extremity by encircling the limb with it above the knee, the erysipelas having crept up the leg thus far. We found frequent bathing of the part with warm spirit and water very agreeable to the feelings of the patient, and it constitutes, probably, with the exception of the nitrate of silver, as good an application as any that can be made use of; a prompt and efficient constitutional treatment, in this form of the disease at least, being, in our judgment, both as regards the throat and face, by far the most momentous and that on which the chief stress should be placed.

We may add, that our friends Dr. Bowen, of North Pitcher, and Dr. Halbert, of Pitcher village, three and seven miles distant from us, had each of them several cases, the epidemic visiting their neighborhoods subsequently to ours. Dr. H. had a greater proportion of cases of the phlegmonous variety of the disease than was observed by us; but the character of the cases met with by both these physicians accorded with the description we have given, and demanded the same plan of treatment.

As a mere guess, we may state that the whole number of cases occurring in the neighborhood, severe and mild, and among practitioners, regular and irregular, might be 200, and the deaths between forty and fifty.

